# The use of recombinant fish parvalbumin Gad c 1 in the characterisation of fish allergic patients

**DOI:** 10.1186/2045-7022-5-S3-P22

**Published:** 2015-03-30

**Authors:** Fátima Cabral Duarte, Ana Célia Costa, Manuel Augusto Pereira Barbosa, Maria Conceição Pereira Santos

**Affiliations:** 1 Immunoallergology Department, Hospital de Santa Maria, Lisbon, Portugal; 2 Clinical Immunology Unit, Instituto de Medicina Molecular, Faculdade de Medicina de Lisboa, Lisbon, Portugal

## Background

IgE mediated allergy to fish is a cause of severe anaphylatic reactions. Parvalbumins are considered major fish allergens and also responsible for cross-reactivity.

## Objective

Evaluation of tolerance acquisition using the recombinant fish parvalbumin (Gad c 1) in a group of fish allergic patients (pts).

## Material and methods

We selected 55 pts (34M, 21F; average age:7.4 years). All had fish allergy, characterized by positive clinical history, skin-prick tests and serum specific IgE to several fish and Gad c 1 (UniCap^®^, Thermo-Fisher). Oral food challenge was performed to evaluate fish tolerance acquisition. Statistical analysis was performed using GraphPad Prisma version 5.0 (GraphPad Software Inc, SD). Two groups were compared using a paired t test Wilcoxon. P values<0.05 were considered significant.

## Results

The 55 fish allergic pts had clinical symptoms for more than one fish species: 43 (78%) pts had skin manifestations, 12 (22%) anaphylaxis, 8 (15%) respiratory and 9 (16%) gastrointestinal symptoms. Pts who suffered anaphylaxis had lower Gad c 1 average values (31 KU/L) than the others (76.7 KU/L); *p*=0.15. 38/55 (69%) pts were intolerant and 17/55 (31%) pts tolerated at least one fish species. Gad c 1 average values were significantly higher (21.6 KU/L) in the intolerant group than in the partially tolerant group (2.7 KU/L); *p*=0.009. 24/38 pts were followed for at least one year, on average 3.7 years. 14/24 pts developed fish tolerance to at least one fish species-Group I (10M, 4F; average age 7 years) and 10/24 were intolerant-Group II (6M, 4F; average age 7 years). Group I showed lower average values (4.6 KU/L) of Gad c 1 than the Group II (25.1 KU/L); *p*=0.0001.

## Conclusions

Lower Gad c 1 values seem to be an important predictive parameter in fish tolerance acquisition but not in predicting clinical symptoms’ severity and might be considered a useful tool in the follow-up of fish allergic patients.

